# Mechanism Analysis of LINC00665 and Its Peptides CIP2A-BP in Hepatocellular Carcinoma

**DOI:** 10.3389/fgene.2022.861096

**Published:** 2022-03-08

**Authors:** Yi-Ran Li, Rui-Qing Zong, Hong-Yan Zhang, Xiao-Yan Meng, Fei-Xiang Wu

**Affiliations:** Department of Intensive Care Medicine, Eastern Hepatobiliary Surgery Hospital, The Third Affiliated Hospital of Naval Medical University, Shanghai, China

**Keywords:** hepatocellular carcinoma, LINC00665, CIP2A-BP, biomarkers, biological information analysis

## Abstract

**Background:** More and more studies show that long non-coding RNAs (lncRNAs) have miniature open reading frames that can be translated into short peptides. Here, we identify the long non-coding gene LINC00665 and its short peptides (CIP2A-BP) in hepatocellular carcinoma (HCC) and explore how they contribute to HCC progression.

**Materials and methods:** First, GSE101728 data were acquired through the Gene Expression Omnibus for identification of differentially expressed genes (DEGs), and gene set enrichment analysis (GSEA) was conducted to find enriched biological pathways. Then, further bioinformatics analysis was carried out on the screened long non-coding genes, and LINC00665 expression was detected in HCC and normal liver samples. The relations between LINC00665 expression, HCC prognosis, and clinical characteristics were studied. Receiver operating characteristic (ROC) analysis was also applied to verify the LINC00665 prediction in HCC prognosis. In addition, pertinent experiments on LINC00665 and CIP2A-BP were also carried out to explore their roles in the progression of HCC.

**Results:** As a result, we screened out 332 DEGs in total, including 130 upregulated and 202 downregulated DEGs. These DEGs were mainly enriched in posttranscriptional regulation of gene expression, RNA processing, nucleolus, and gene silencing biological pathways. In addition, we found that LINC00665 was increased in HCC samples, which substantially indicated its poor prognosis. Compared with normal tissues, LINC00665 had higher expression in the pathological stages III and IV, tumor-free groups, people no more than 60 years old, and stages T3, T4, N0, N1, and M1. ROC curve indicated that the variable INC00665 had certain accuracy in predicting overall survival (OS). Moreover, in functional experiments, LINC00665 knockdown could significantly decrease HCC cell proliferation, migration, and invasion, while overexpressed CIP2A-BP could markedly increase HCC cell proliferation, invasion, and migration.

**Conclusion:** Our findings not only disclose a unique mechanism by which CIP2A-BP encoded by LINC00665 promotes HCC carcinogenesis but suggest that these long non-coding genes and short peptides could be used as biomarkers for HCC diagnosis and prognosis and new targets for HCC therapy.

## Background

Liver cancer (LC) is an extremely harmful malignant tumor in surgical diseases, which is generally divided into primary and secondary types (Sia et al., 2017). The former originates from the liver epithelial or mesenchymal tissues, including hepatocellular carcinoma (HCC), cholangiocarcinoma, and mixed liver cancer (Bosch et al., 1999; Bosch et al., 2004). The latter is formed by the metastasis of malignant tumors from the stomach, pancreas, biliary tract, and others to the liver (Ananthakrishnan et al., 2006). Reports from the International Agency for Research on Cancer show that during 2020, more than 900,000 people worldwide had been diagnosed with LC; the incidences of men and women were 6.3 and 3.0%, respectively, and the number of deaths exceeded 830,000 (Rodriguez-Acevedo et al., 2020). Currently, clinical investigations have found that viral hepatitis, food contaminated by *Aspergillus flavus* and its mycin, drinking alcohol, water pollution, chemical carcinogens, and genetic factors are all risk factors for LC occurrence (Yuen and Wong, 2020). Due to the hidden incidence, rapid invasive growth, and high mortality, the treatment and prognosis of patients are relatively poor (Jiao et al., 2019). Therefore, to prolong the survival time of patients and improve their life quality, there is an urgent need for the emergence of safe and effective anticancer technologies.

Long non-coding RNA (lncRNA) is a kind of non-coding RNA which is transcribed in most eukaryotic genomes (Wright, 2014). Studies have found that lncRNA can perform its functions by binding to DNA/RNA or proteins in the regulation of multiple biological processes like genome imprinting, X chromosome silencing, chromatin modification, nuclear transport, transcription activation, and interference (Yang et al., 2015). In addition, lncRNA is also associated with human cell differentiation, growth, reproduction, gender regulation, aging, and many diseases (Shi et al., 2013). Presently, there have been many studies on lncRNA and LC. For instance, Feng et al. (2019) analyzed the role of lncRNA PCNAP1 in the replication of hepatitis B virus (HBV) and found that lncRNA PCNAP1 enhanced HBV replication by regulating the miR-154/PCNA/HBV axis, thereby driving the occurrence of LC. Wu et al. (2019) demonstrated that high-expressed lncRNA CEBPA-AS1 in LC tissues promoted the size of tumors and the survival activity of cancer cells. However, further exploration on the mechanism of lncRNA in LC is needed.

LINC00665 belongs to the lncRNA class, and it has been reported to have certain functions in multiple diseases, such as triple-negative breast cancer (Guo et al., 2020) and osteosarcoma (Zhang et al., 2020). Chen et al. (2020) observed highly expressed LINC00665 in prostate cancer tissues, and it could significantly promote the expression of SND1 by inhibiting miR-1224-5p through bioinformatics and functional experimental analysis. As a short peptide encoded by LINC00665, CIP2A-BP is also found to have an indispensable role in many cancers. For example, CIP2A-BP can interact with CIP2A to inhibit the PI3K/Akt/NFkB pathway in triple-negative breast cancer, thereby affecting the survival activity of cancer cells (Guo et al., 2020). Here, we will focus on the specific mechanism of LINC00665 and CIP2A-BP in HCC.

## Materials and Methods

### Public Database

The Cancer Genome Atlas (TCGA, https://www.cancer.gov/about-nci/organization/ccg/research/structural-genomics/tcga) database (Hutter and Zenklusen, 2018) is a groundbreaking cancer genome research project, covering more than 20,000 primary cancers and matching normal samples. Gene Expression Omnibus (GEO, https://www.ncbi.nlm.nih.gov/geo/) (Chicco, 2022) is the world’s biggest and most comprehensive public gene data collection, encompassing nearly all illnesses and incorporating gene expression, mutation, alteration, and other data. We screened the HCC chip expression data from the TCGA and GEO databases and determined LINC00665 as an HCC clinical diagnostic marker for follow-up research.

### Acquisition of Microarray Data and Filtering of Differentially Expressed Genes

We downloaded the GSE101728 dataset from the GEO database, which contained 7 HCC and 7 adjacent tumor-free samples. After that, DEG analysis was performed on the genes in these samples by the GEO2R (https://www.ncbi.nlm.nih.gov/geo/geo2r/) tool, and the filtering condition for upregulated DEGs was set to a fold change (FC) > 2, and that for downregulated DEGs was FC < 0.5, both satisfying *p <* 0.001.

### Gene Set Enrichment Analysis

GSEA (http://software.broadinstitute.org/gsea/index.jsp) is a computational approach for identifying potential biological pathways based on RNA expression profiles. After the DEGs were screened out, we explored the Gene Ontology (GO) enrichment of these DEGs in the Molecular Signatures Database (MSigDB) database (https://www.gsea-msigdb.org/gsea/msigdb) through the GSEA method, and results with *p <* 0.05 had statistical significance.

### Expression Analysis of LINC00665

Next, the expression level of LINC00665 was verified in HCC and normal samples based on the GSE101728 dataset in the GEO database, and then the relevant expression levels of LINC00665 were analyzed in HCC normal tissues and tumor tissues in the TCGA database.

### Prognostic Analysis of LINC00665

We analyzed the relationship between LINC00665 and the overall survival (OS) time of HCC patients in the Kaplan–Meier (KM) plotter website and showed the hazard ratio (HR) with 95% confidence intervals (CI) and the log-rank *p-*value. A *p* value less than or equal to 0.05 was considered statistically significant. By analyzing the prognosis of patients, it was helpful to understand the impact of LINC00665 on HCC short-term and long-term prognosis.

### Clinical Characteristic Analysis of LINC00665

Afterward, we used normal tissues as a control to verify the expression of LINC00665 in HCC patients with different conditions in the TCGA database, including pathologic stage (stages I–IV), tumor status (tumor-free, with tumor), age (≤60, >60), T stage (T1–T4), N stage (N0 and N1), and M stage (M0 and M1), and the final results were displayed using box plots.

### Diagnostic Efficiency Evaluation for Hepatocellular Carcinoma

The receiver operating characteristic (ROC) curve, also known as the sensitivity curve, is used to describe the inherent truthfulness of diagnostic tests. This time, we drew the ROC curve about the OS of HCC patients based on the GraphPad Prism tool, evaluated the correlation between LINC00665 expression and OS, and calculated the relevant area under the curve (AUC) values and 95% CI to explore the clinical diagnostic value. A *p* value <0.05 was selected as the cutoff criteria.

### Cell Culture and Transfection

Human LC cells (MHCC97H and SNU387) were obtained from the Cell Bank of the Chinese Academy of Sciences (Shanghai, China), and cultured in RPMI1640 medium (Gibco, United States) containing 10% fetal bovine serum (Hyclone, United States). After that, the environment in the culture medium was maintained at 37°C containing 5% CO_2_. Then, with vector as a control, LINC00665 (over-LINC00665, si-LINC00665 #1, and si-LINC00665 #2) and CIP2A-BP (over-CIP2A-BP) were transfected. Finally, based on the transfection efficiency, si-LINC00665 #2 and over-CIP2A-BP were chosen for the next study.

### Quantitative Real-Time Polymerase Chain Reaction (qRT-PCR)

Total RNA was extracted from MHCC97H and SNU387 cells by TRIzol (Invitrogen, CA, United States), and then complementary DNA (cDNA) was synthesized by using an miRNA First-Strand cDNA Synthesis Kit (Tiangen, Beijing, China). Subsequently, qRT-PCR was conducted by using a SuperReal PreMix Plus SYBR Green Kit (Tiangen, Beijing, China) and miRcute Plus miRNA qPCR Detection Kit (Tiangen, Beijing, China). Finally, the 2^−ΔΔCT^ equation method was used to compute potential gene expression.

### Cell Proliferation Assay

In the cell proliferation experiment, we first seeded the cells into a 96-well plate at a density of 1 × 10^3^ and then injected a certain amount of CCK-8 (Cell Count Kit-8; Beyotime Biotechnology, Shanghai, China) solution into each well at 0, 24, 48, 72, and 96 h. Finally, si-NC was used for comparison, and the relative cell numbers after LINC00665 knockdown and overexpression of CIP2A-BP were calculated by microscopy to determine cell proliferation activity.

### Transwell Assay

For cell migration and invasion, we used Transwell methods. First, after incubating the MHCC97H and SNU387 cell lines for 24 h, the excess cells on the surface of the upper chamber were wiped off. Then, the cells in the lower chamber were stained with 0.5% crystal violet (Sigma-Aldrich, United States), the five fields of view were randomly selected to take pictures, and the average number of the stained cells per field was counted to observe the migration ability. Next, MHCC97H and SNU387 cell lines were placed in the upper chamber coated with 2 mg/ml Matrigel for the detection of invasion ability.

### Statistical Analysis

For statistical analyses, GraphPad Prism 5 (GraphPad Software, United States) was used. Data from three independent experiments were exhibited as mean ± standard deviation (SD). For parametric data, unpaired, 2-tailed Student’s t-tests were used, and for non-parametric data, a 2-sided Mann–Whitney *U* test was used. Differences with *p <* 0.05 indicated statistical significance.

## Results

### Identified Results of Differentially Expressed Genes

**FIGURE 1:**
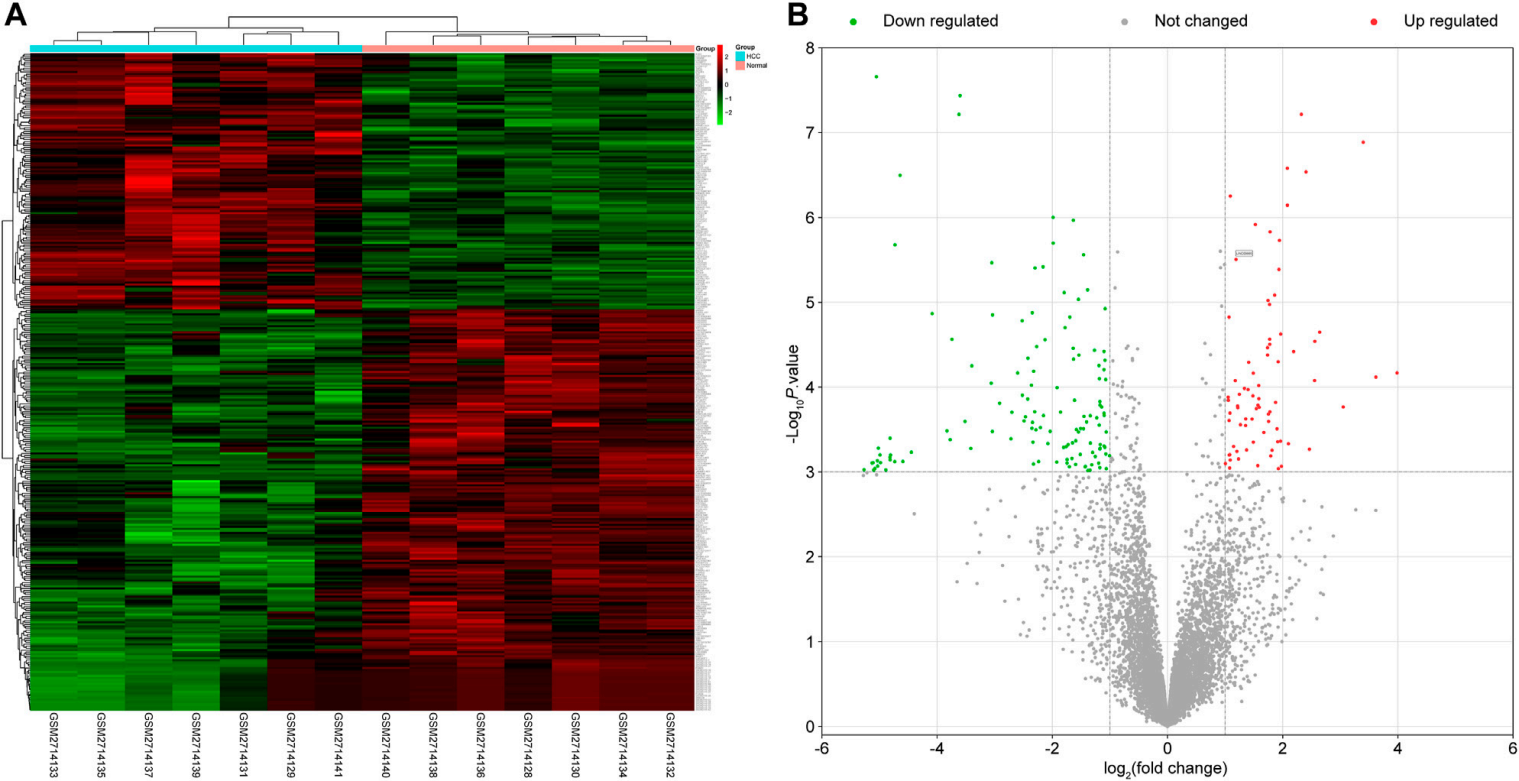
Analysis results of 332 DEGs. **(A)** Heat map. The blue part represents HCC samples, and the pink part represents control samples. **(B)** Volcano map. Red represents DEGs that are upregulated, green represents DEGs that are downregulated, and gray represents not changed genes.

After analyzing the lncRNAs in the GSE101728 data set by the GEO2R method, we obtained 332 DEGs, of which 130 were upregulated and 202 were downregulated. Furthermore, the top 10 upregulated DEGs were LOC344887, LUCAT1, HOXA-AS2, PTTG3P, LINC01136, LOC254896, PVT1, DUXAP8, CELSR3-AS1, and MIR4435-2HG. The top 10 downregulated DEGs were MIR6829, LINC00977, TTTY14, MIR6131, LINC00635, LINC01405, MIR29C, RAB11B-AS1, FCGR2C, and DNM3OS. The distribution of these DEGs in the sample was shown by a heat map and a volcano map (Figures 1A,B).

### Enriched GO Terms in Gene Set Enrichment Analysis

**FIGURE 2:**
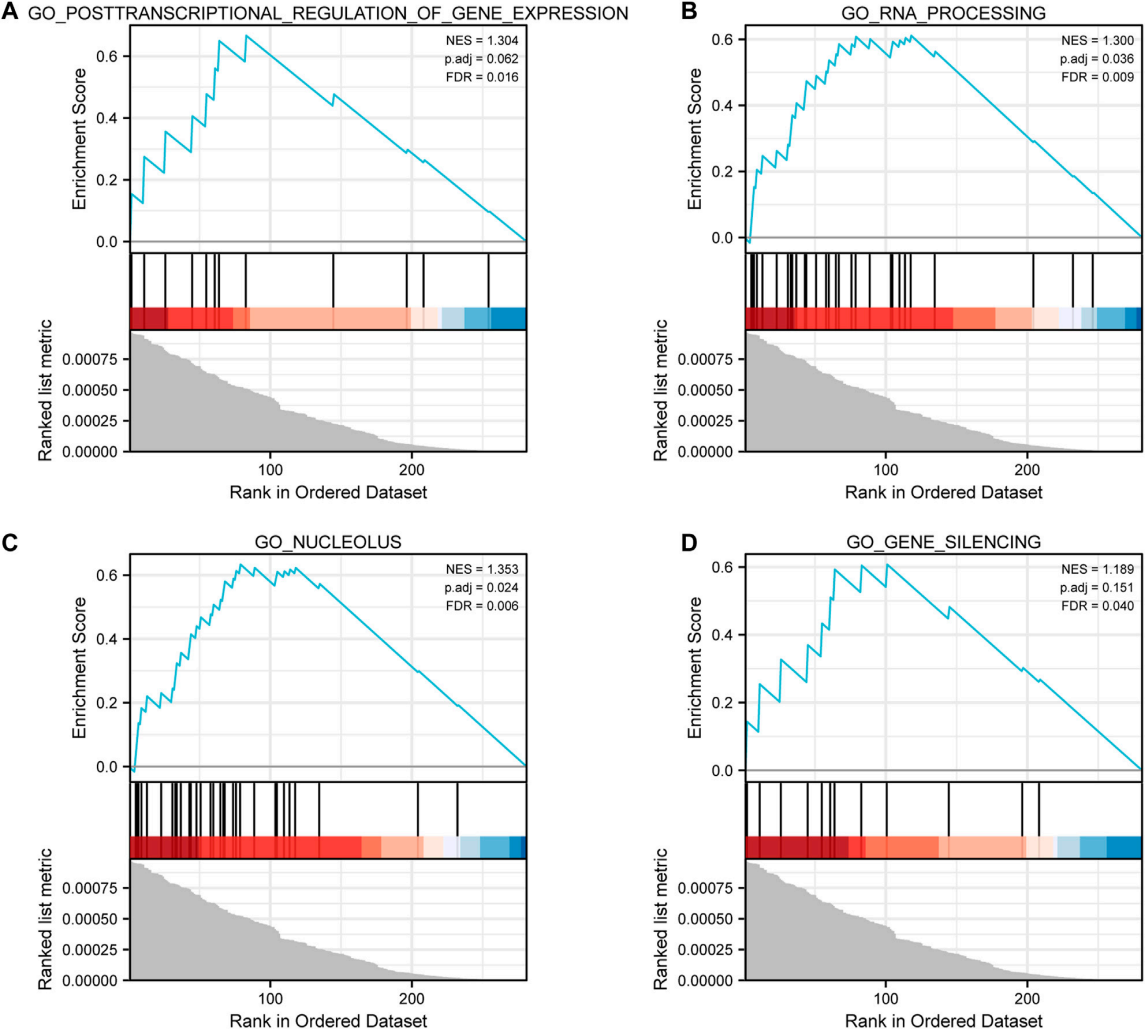
GSEA results. **(A)** Enrichment plot for posttranscriptional regulation of gene expression. **(B)** Enrichment plot for RNA processing. **(C)** Enrichment plot for the nucleolus. **(D)** Enrichment plot for gene silencing.

According to GSEA, we got 4 GO-related enrichment items, namely, posttranscriptional regulation of gene expression (normalized enrichment score, NES = 1.304, *p =* 0.062, false discovery rate, FDR = 0.016), RNA processing (NES = 1.300, *p =* 0.036, FDR = 0.009), nucleolus (NES = 1.353, *p =* 0.024, FDR = 0.006), and gene silencing (NES = 1.189, *p =* 0.151, FDR = 0.040, Figures 2A–D).

### LINC00665 Expressions in Public Databases

**FIGURE 3:**
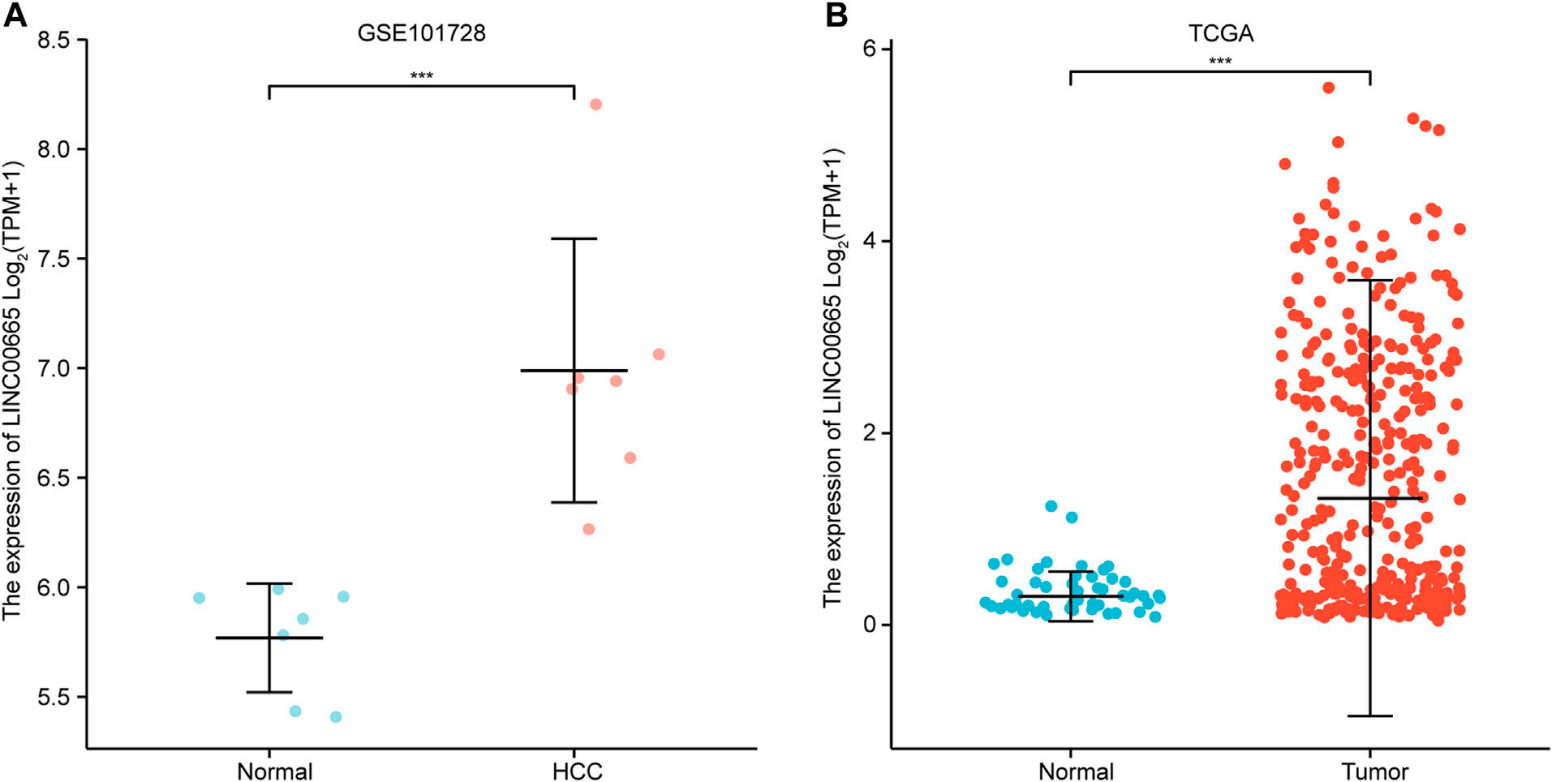
Expression analysis of LINC00665 in HCC. **(A)** GSE101728 data set. **(B)** TCGA database. ***p < 0.001.

In the GSE101728 data set, compared with normal tissues, LINC00665 was upregulated in HCC tissues (Figure 3A). Similarly, the analysis of the TCGA database demonstrated that LINC00665 expression in the tumor tissues was also significantly upregulated (Figure 3B).

### Clinical Prognosis of LINC00665

**FIGURE 4:**
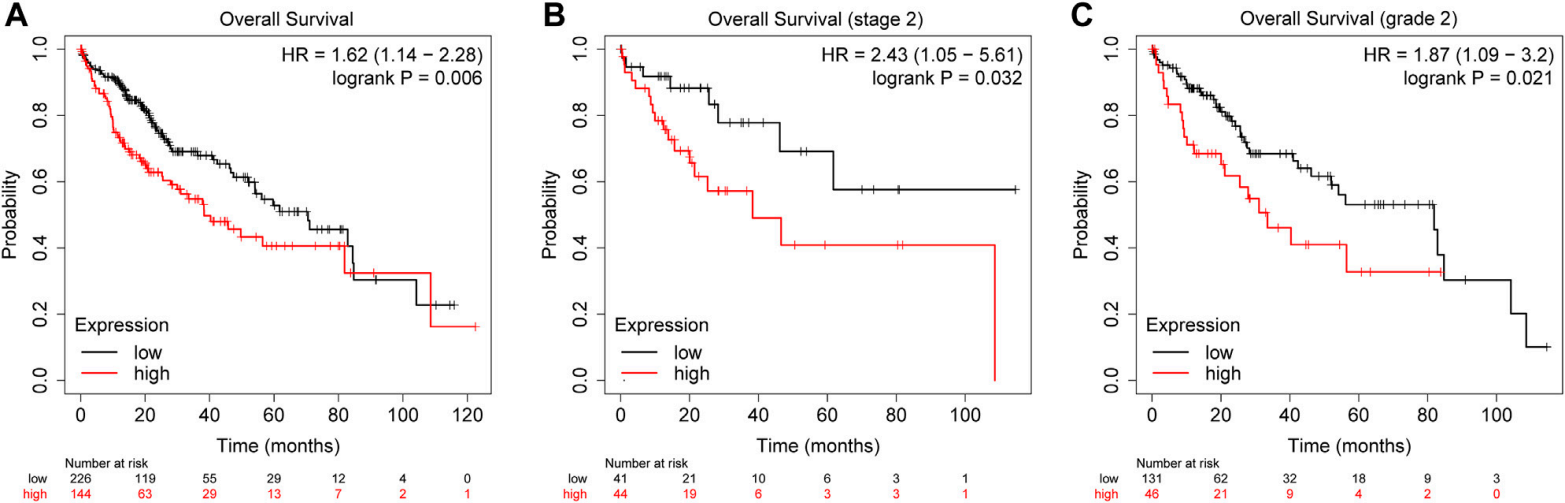
KM survival curve. Red represents the high expression, and black represents is the low expression. **(A)** OS, 370 samples. **(B)** OS stage 2, 85 samples. **(C)** OS grade 2, 177 samples.

To explore the role of LINC00665 in HCC prognosis, we plotted the KM survival curve to show the effect of differentially expressed level LINC00665 on the OS of patients. In OS (HR = 1.62, log-rank *p =* 0.006), OS for stage 2 (HR = 2.43, log-rank *p =* 0.032), and OS for grade 2 (HR = 1.87, log-rank *p =* 0.021), the high expression of LINC00665 reduced the patients’ survival probability and shortened the patients’ time (Figures 4A–C).

### LINC00665 Expressions in Hepatocellular Carcinoma Patients With Different Clinical Characteristics

**FIGURE 5:**
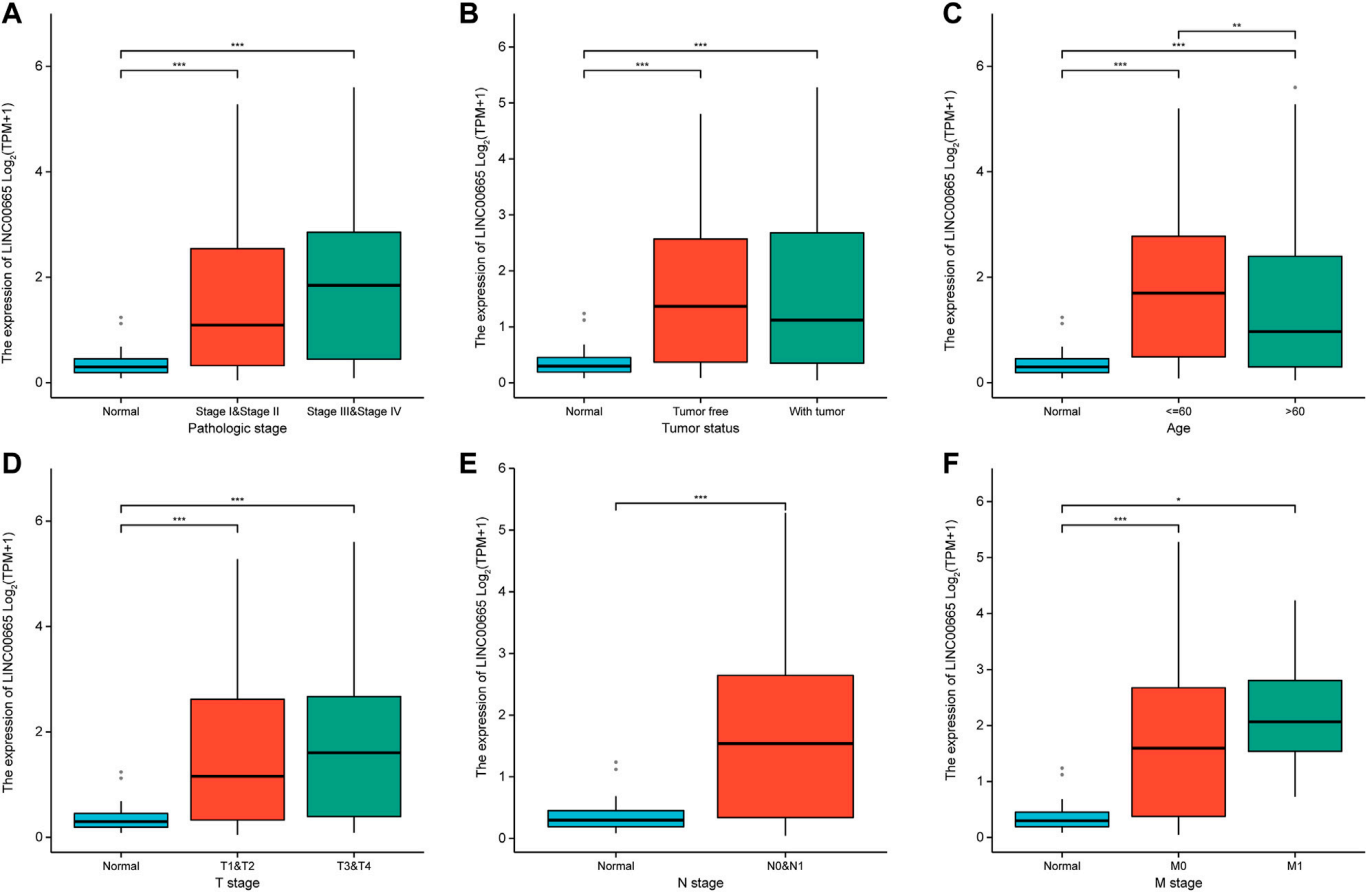
Box plot indicated the expression levels of LINC00665 in HCC patients with different clinical characteristics. **(A)** Pathologic stage. **(B)** Tumor status. **(C)** Age. **(D)** T stage. **(E)** N stage. **(F)** M stage. *p < 0.05, **p < 0.01, ***p < 0.001.

Figures 5A–F show the relative levels of LINC00665 in different clinical stages of HCC patients. In comparison with normal groups, LINC00665 had a higher expression in the pathological stages III and IV, tumor-free, ≤60 group, and stages T3, T4, N0, N1, and M1. Then, it could be concluded that with the further development of the tumor, the expression of LINC00665 gradually increased, which was closely related to clinical diagnosis.

### Analysis of ROC Curve

**FIGURE 6:**
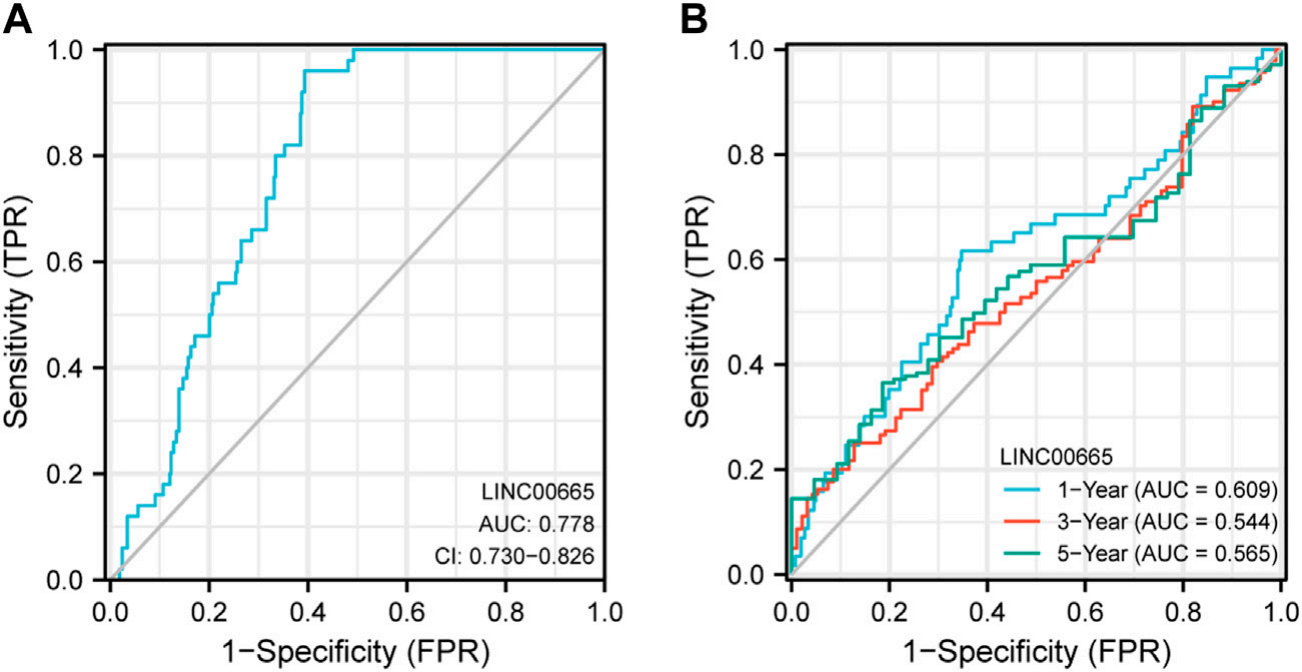
ROC curve analysis. The ordinate represents sensitivity, and the abscissa represents 1-specificity. **(A)** OS. **(B)** The blue line represents the 1-year OS, the red line is the 3-year OS, and the green is the 5-year OS.

The ROC curve was adopted to assess the sensitivity of LINC00665 to the prognosis of HCC patients. According to Figure 6A, it was found that the variable LINC00665 had certain accuracy in predicting OS (AUC: 0.778, CI: 0.730–0.826). In addition, the results in Figure 6B indicated that the AUCs of 1, 3, and 5 years were 0.609, 0.544, and 0.565, respectively, indicating that the predictive ability of the variable LINC00665 had low accuracy.

### Knockdown of LINC00665 Expression Inhibited the Proliferation, Migration, and Invasion of Hepatocellular Carcinoma Cells

**FIGURE 7:**
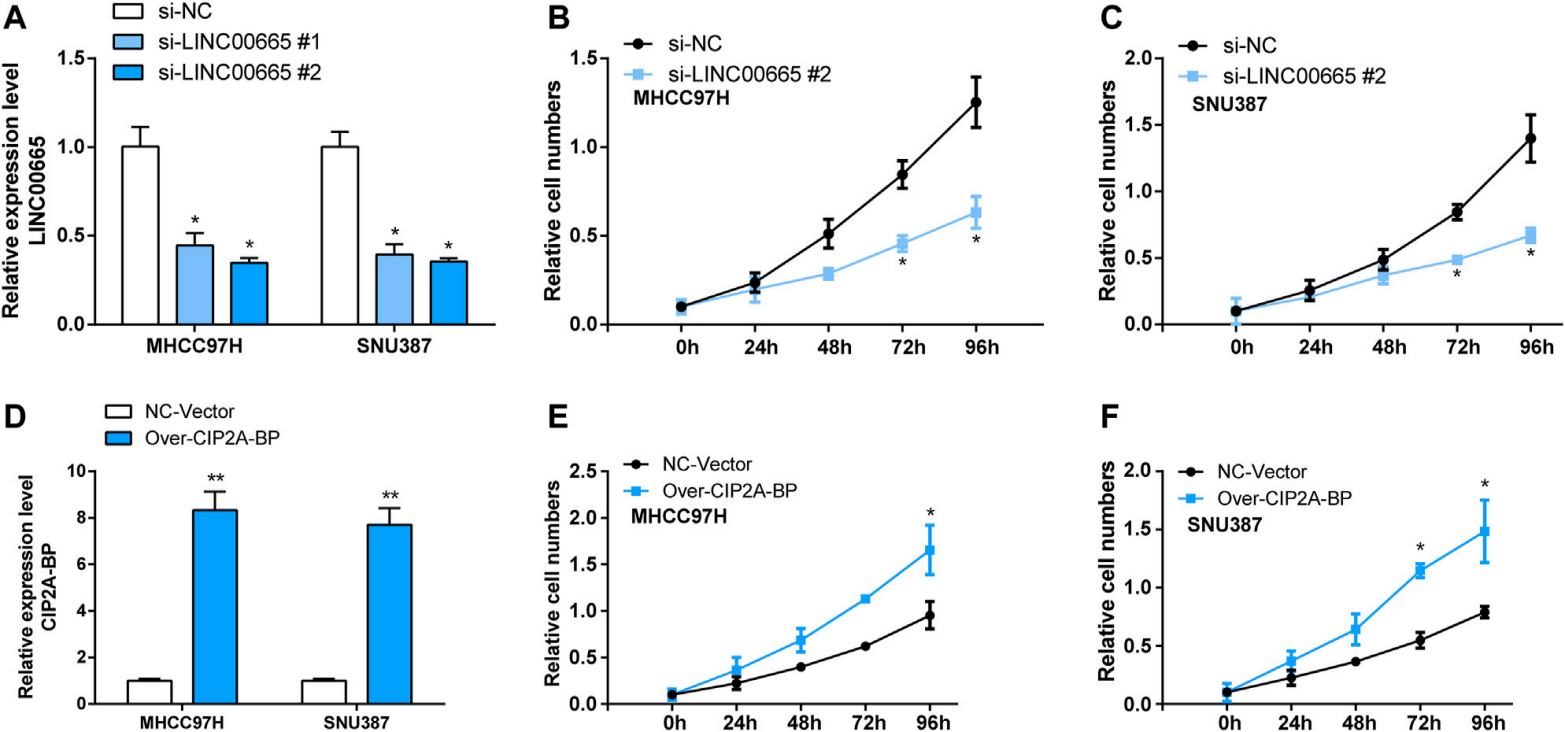
Knockdown LINC00665 inhibited the proliferation of HCC cells, and overexpression of CIP2A-BP promoted proliferation of HCC cells. **(A)** Knockdown transfection of LINC00665 in HCC cells (si-LINC00665 #1 and si-LINC00665 #2). **(B)** The effect of si-LINC00665 #2 on the proliferation of MHCC97H cells. **(C)** The effect of si-LINC00665 #2 on the proliferation of SNU387 cells. **(D)** Overexpression transfection of CIP2A-BP in HCC cells. **(E)** The effect of over-CIP2A-BP on the proliferation of MHCC97H cells. **(F)** The effect of over-CIP2A-BP on the proliferation of SNU387 cells. *p < 0.05, **p < 0.01.

**FIGURE 8:**
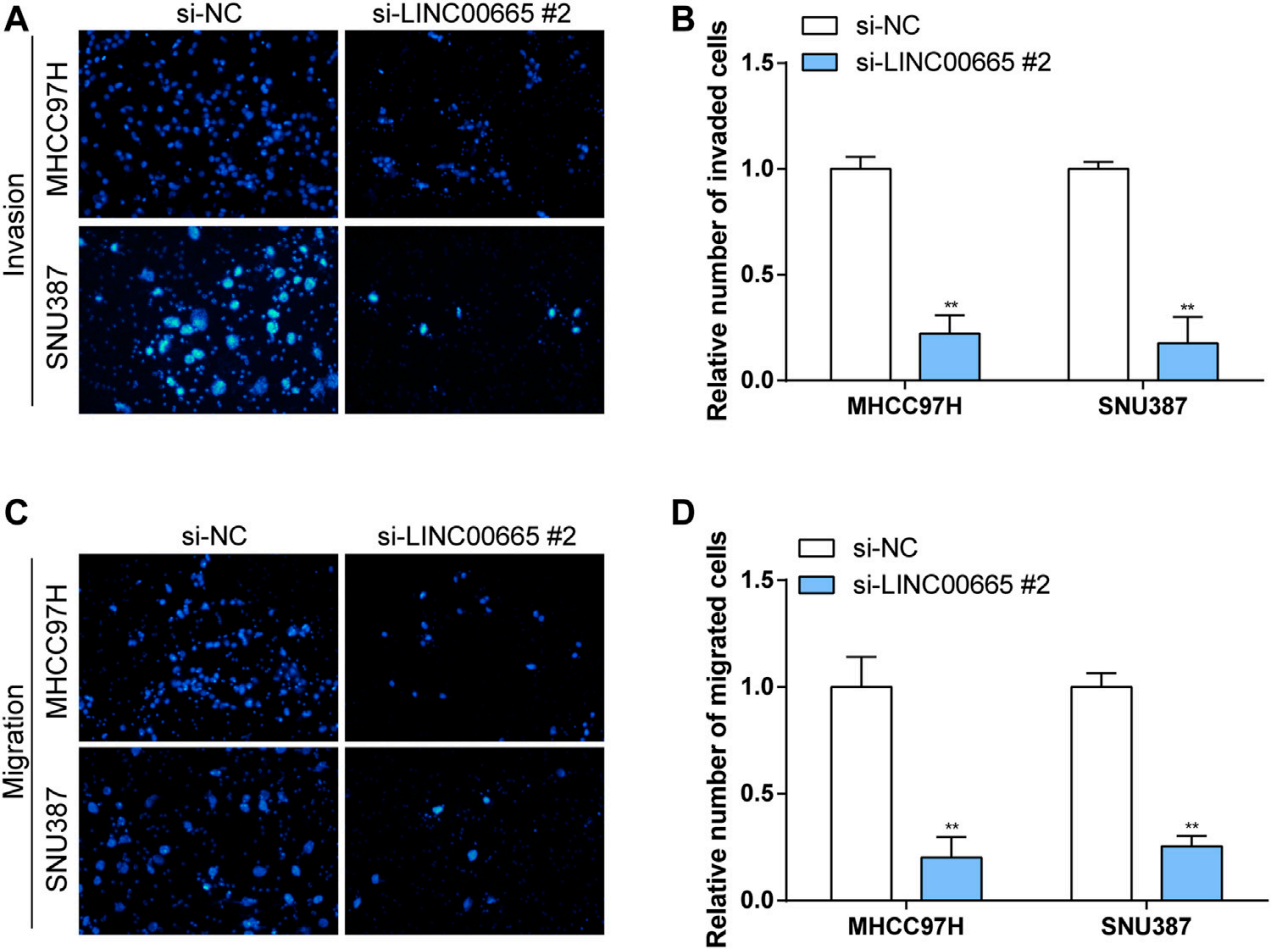
Knockdown of LINC00665 expression inhibited the migration and invasion of HCC cells. Knockdown of LINC00665 expression significantly inhibited the invasive and migratory abilities in MHCC97H and SNU387 cells. **(A,C)** Fields of view observed by the microscope. **(B,D)** Histograms of invasion and migration results. **p < 0.01.

To explore the specific functional role of LINC00665 in HCC, we first knocked down LINC00665 in MHCC97H and SNU387 with si-LINC00665 #1 and si-LINC00665 #2 (Figure 7A). In the results of siRNA transfection, the knockdown effect of si-LINC00665 #2 was more evident, which could effectively inhibit the relative expression level of LINC00665 in HCC cells. Thus, si-LINC00665 #2 was applied for the next experiments. Then, in the CCK-8 experiment, compared with si-NC, the cell proliferation activities of MHCC97H and SNU387 transfected with si-LINC00665 #2 were significantly inhibited (Figures 7B,C). Furthermore, we also observed that the HCC cell migration and invasion were also significantly reduced after the knockdown of LINC00665 expression (Figures 8A–D).

### Overexpression of CIP2A-BP Promoted Proliferation, Invasion, and Migration of Hepatocellular Carcinoma Cells

**FIGURE 9:**
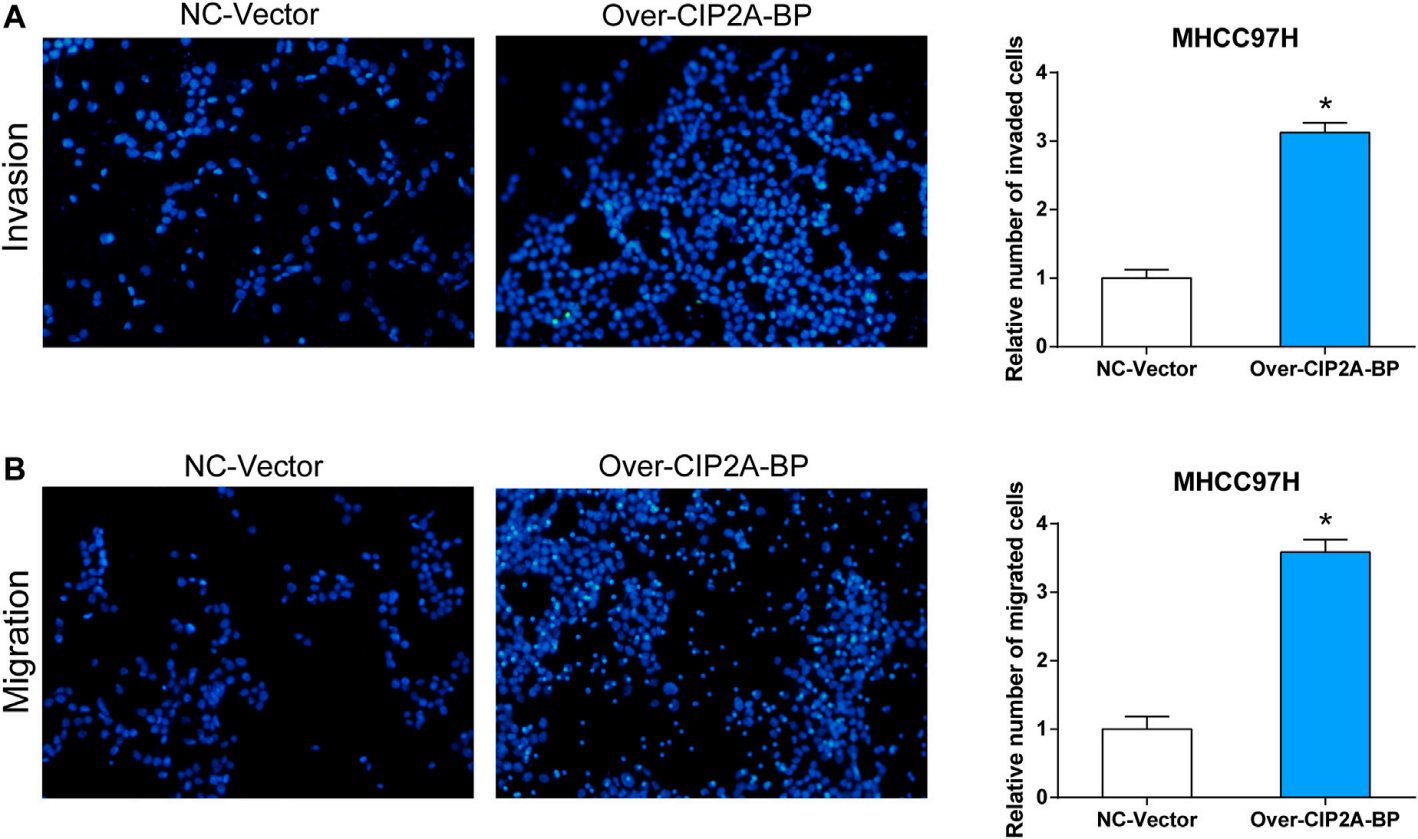
Overexpression of CIP2A-BP promoted invasion and migration of HCC cells. The effect of over-CIP2A-BP on the **(A)** invasion and **(B)** migration ability of MHCC97H cells. *p < 0.05.

In addition, we also verified the effect of overexpressed CIP2A-BP on the activities of HCC cells. According to the results in Figure 7E, it was found that the contents of CIP2A-BP in MHCC97H and SNU387 cells were significantly increased compared with NC vector. The experimental results of CCK-8 proved that over-CIP2A-BP increased the relative cell number of HCC (Figures 7F,G), and Transwell also showed that under the microscope observation, the invasion and migration of MHCC97H cells transfected with over-CIP2A-BP were significantly increased (Figures 9A,B). From this, we could conclude that the overexpression of CIP2A-BP was positively related to the cell proliferation, invasion, and migration of HCC cells.

## Discussion

A growing number of studies show that abnormally expressed lncRNAs are found in various human cancers, and they may influence multiple biological processes like cell cycle progression, apoptosis, cell migration, and invasion (Luo et al., 2017; Li et al., 2018; Wang et al., 2020). In addition, the molecular mechanisms on how lncRNAs exert their biological roles are omnifarious and intricate. During these mechanisms, the function of small open reading frames on their translation products (short peptides) has been largely ignored due to the difficulty in identifying functional small open reading frames (Andrews and Rothnagel, 2014; Ruiz-Orera and Albà, 2019). Here, we aimed to investigate the molecular mechanism among lncRNAs, peptides, and HCC.

In the present study, we screened the non-coding genes in the data set GSE101728 containing 7 HCC samples and 7 normal samples from adjacent tissues. In the identified non-coding gene, we determined to study the regulatory mechanisms of the lncRNA LINC00665 in LC because there have been reports on LINC00665 and human diseases. Later, we detected LINC00665 expressions in GSE101728 and TCGA datasets, and the results exhibited that LINC00665 was upregulated in HCC groups, which was consistent with what the previous research reported. Also, LINC00665 was knocked down in HCC cell lines to observe the effect of its decrease on the HCC cell proliferation, migration, and invasion. The experimental results exhibited that LINC00665 knockdown significantly reduced the cell activities in HCC.

Moreover, we detected the short peptide CIP2A-BP produced by LINC00665 and investigated its functions in HCC. We overexpressed CIP2A-BP in HCC cell lines and found that overexpressed CIP2A-BP significantly increased the cell number in CCK-8 experiments. Transfection experiments also showed an evident increase in the number of MHCC97H cells in CIP2A-BP, so MHCC97H was used for the following experiments. Also, the experimental findings demonstrated increased cell invasion and migration under microscope observation. From these, we can conclude that both LINC00665 and CIP2A-BP have oncogenic roles in HCC development.

Moreover, to investigate the biological information of HCC, we screened the DEGs from the data set of GSE101728, in which we obtained 332 DEGs, including 130 upregulated and 202 downregulated DEGs. In the TCGA public database, LINC00665 had a high level in HCC tissues. In addition, we also predicted the prognostic effect of LINC00665 in liver cancer patients through the KM survival curve and ROC analysis. It was demonstrated whether in the staging or grading of patients with LC, the highly expressed LINC00665 had a lower survival time and poor prognosis in HCC patients. ROC curve indicated that the variable LINC00665 had certain accuracy in predicting OS. Then, the relation between LINC00665 and clinical characteristics was also investigated. Compared with control groups, LINC00665 had higher expressions in the pathological stages III and IV, tumor-free group, people no more than 60 years old, and stages T3, T4, N0, N1, and M1.

In the functional analysis, GSEA was also performed on DEGs. The results indicated that they were mainly enriched in posttranscriptional regulation of gene expression, RNA processing, nucleolus, and gene silencing biological pathways. Currently, there have been studies on these pathways and HCC progression. For instance, nucleoli usually appear as single or more homogeneous spherical bodies and are the most prominent structures in eukaryotic interphase nuclei. The main function of the nucleolus is to synthesize ribosomes and RNA (rRNA) (Scheer and Hock, 1999; Boisvert et al., 2007). Ribosome biogenesis is an important biological process that controls the rate of protein synthesis in cells (Leary and Huang, 2001; Gupta and Santoro, 2020). Abnormal ribosome synthesis has been linked to cancer and malignant transformation in a growing number of studies. Yu et al. (2015) created a high-resolution map of histone modification marks at rDNA in human liver cancer cells, providing new evidence for chromatin-mediated rDNA regulation in LC. As for gene silencing, it represents a process in eukaryotes about the recognition and clearance of abnormal RNA from cells induced by double-stranded RNA (Meister and Tuschl, 2004; Duchaine and Fabian, 2019). Presently, numerous articles have demonstrated that gene silencing is related to HCC. For example, Wei et al. (2010) explored the silencing effect of aldo–keto reductase family 1 member B10 gene on the proliferation and apoptosis of HCC cells.

In conclusion, LINC00665 and CIP2A-BP have oncogenic functions in HCC development, and they could be new therapeutic targets and prognostic biomarkers for HCC treatment. In addition, these data emphasize that lncRNA-encoded peptides can encode functional peptides with clinical and therapeutic relevance. Nevertheless, there are several limitations in this article. We studied the role of LINC00665 and CIP2A-BP in HCC. HCC is a malignant disease with extensive molecular heterogeneity. Therefore, in the future, it would be worth exploring other potential regulatory mechanisms of CIP2A-BP in other HCC subtypes. Also, investigating CIP2A-BP upstream regulation would help clarify the comprehensive tumor-suppressive roles of CIP2A-BP. A deeper understanding of LINC00665 and CIP2A-BP should be explored.

## Data Availability

The original contributions presented in the study are included in the article/Supplementary Material; further inquiries can be directed to the corresponding author.
